# Translocon Remodeling Modulates Ribosomal Frameshifting and the Maturation of the Sindbis Virus Structural Polyprotein

**DOI:** 10.64898/2026.07.27.741003

**Published:** 2026-08-04

**Authors:** Antonio Bonifasi, Gayani Apsara Ranasinghe, Ashish R. Jhangiani, Venkatesh P Thirumalaikumar, Brianna Corman, J. Paul Robinson, Suchetana Mukhopadhyay, Jonathan P. Schlebach

## Abstract

Like other enveloped RNA viruses, alphaviruses synthesize and assemble their envelope glycoproteins at the endoplasmic reticulum (ER) membrane. There, protein-conducting channels called translocons provide nascent proteins access to the membrane and allow them to fold into their correct shapes. We previously showed that a hydrophobic segment in the Sindbis virus structural polyprotein forms cotranslational interactions with the translocon that enhance −1 programmed ribosomal frameshifting (−1PRF), a recoding event that regulates polyprotein biogenesis. Recent discoveries concerning translocon remodeling suggest this segment, which corresponds to the second transmembrane domain of the E2 protein, could serve as a signal that recruits the multipass translocon (MPT). Here, we show that knocking out certain components of the MPT increases −1PRF efficiency. These differences in recoding coincide with changes in the membrane topology of the nascent polyprotein and in its downstream proteolytic processing in a manner that ultimately reduces viral fitness. Together, our results indicate that −1PRF and spike protein maturation in alphaviruses is tuned by the dynamic remodeling of the ER translocon. Such coupling could allow polyprotein biogenesis to adapt to different stages of viral replication and to distinct host or vector environments.

## Full Text Availability

The license terms selected by the author(s) for this preprint version do not permit archiving in PMC. The full text is available from the preprint server.

